# A global dataset of sequence, diversity and biosafety recommendation of arbovirus and arthropod-specific virus

**DOI:** 10.1038/s41597-023-02226-8

**Published:** 2023-05-19

**Authors:** Ying Huang, Shunlong Wang, Hong Liu, Evans Atoni, Fei Wang, Wei Chen, Zhaolin Li, Sergio Rodriguez, Zhiming Yuan, Zhaoyan Ming, Han Xia

**Affiliations:** 1 Key Laboratory of Highly pathogenic Viruses and Biosafety, Wuhan Institute of Virology, Chinese Academy of Sciences, Wuhan, 430071 China; 2 University of Chinese Academy of Sciences, Beijing, 100049 China; 3 School of Life Sciences and Medicine, Shandong University of Technology, Zibo, 255049 China; 4 Department of Microbiology and Immunology, University of Texas Medical Branch, Galveston, 77551 USA; 5 School of Computer and Computing Science, Hangzhou City University, Hangzhou, 310015 China; 6 Hubei Jiangxia Laboratory, Wuhan, 430207 China

**Keywords:** Genetic databases, Virology

## Abstract

Arthropod-borne virus (arbovirus) and arthropod-specific virus (ASV) are viruses circulating amongst hematophagous arthropods that are broadly transmitted in ecological systems. Arbovirus may replicate in both vertebrates and invertebrates and some are known to be pathogenic to animals or humans. ASV only replicate in invertebrate arthropods yet they are basal to many types of arboviruses. We built a comprehensive dataset of arbovirus and ASV by curating globally available data from the Arbovirus Catalog, the arbovirus list in Section VIII-F of the Biosafety in Microbiological and Biomedical Laboratories 6th edition, Virus Metadata Resource of International Committee on Taxonomy of Viruses, and GenBank. Revealing the diversity, distribution and biosafety recommendation of arbovirus and ASV at a global scale is essential to the understanding of potential interactions, evolution, and risks associated with these viruses. Moreover, the genomic sequences associated with the dataset will enable the investigation of genetic patterns distinguishing the two groups, as well as aid in predicting the vector/host relationships of the newly discovered viruses.

## Background & Summary

Generally, there are two major groups of viruses that circulate in hematophagous arthropods: arthropod-borne virus (arbovirus) and arthropod-specific virus (ASV) (or arthropod-only virus). These viruses are diverse in taxonomy and may have unsegmented or segmented (two to twelve) genomes.

Arbovirus are transmitted by hematophagous arthropods such as mosquitoes, ticks, sandflies, and other vectors. Arbovirus can be pathogenic to either animals or humans, such as the mosquito-borne dengue virus, tick-borne Crimean-Congo haemorrhagic fever virus, and midge-borne bluetongue virus. These pathogenic viruses replicate in both vertebrates and invertebrates. With the progression of factors including climate change, urbanization, increased international travel and trade, arbovirus continue to emerge and re-emerge worldwide, which poses a serious challenge to global public health^1^.

Studies over the past decade have established that arthropods do harbor a rich and diverse group of ASV. These ASV naturally infect hematophagous arthropods and replicate both *in vivo* and *in vitro* in these arthropods. However, they are inherently unable to replicate in the vertebrates and their respective cells^1^. Some of these ASV have been classified as members of classical viral families that are traditionally associated with arbovirus, including but not limited to the families *Flaviviridae*, *Togaviridae*, *Reoviridae*, *Peribunyaviridae, Nairoviridae, and Phleboviridae*. It is important to note that viruses identified as ASV may in some cases originate from arthropod commensal fungi/bacteria, and this condition is currently difficult to distinguish^2^. There are two views on the relationship between ASV and arbovirus: (1) ASV are basal to many types of arbovirus based on phylogenetic analysis^3–5^, and (2) ASV are interleaved within arbovirus phylogenies^2,6^. Moreover, both *in vitro* and *in vivo* studies have indicated that ASV may affect the vector competence of arthropods by direct competition with arbovirus or indirectly affecting arthropod physiology, a strategy that can be applied in developing novel approaches for arboviral disease or vector control^3^.

The American Committee on Arthropod-Borne Viruses (ACAV) published the first edition of “Catalogue of Arthropod-borne Viruses of the World (Arbovirus Catalog)” in 1967^7^. The 3^rd^ edition was published in 1985, and it was the last printed version. Currently, the Arbovirus Catalog is maintained by the Centers for Disease Control and Prevention (CDC) on its website (https://wwwn.cdc.gov/arbocat/), and it includes around 537 distinct viruses consisting of both arbovirus and other vertebrate virus (filoviruses, hantaviruses, and arenaviruses). This website records the meta information of each virus including name, original source, method of isolation, virus properties, antigenic relationship, and other factors, but without genetic information such as viral sequence, and whether the genome is segmented or not. Moreover, with classic virus isolation methods and the widespread utilization of deep sequencing and metagenomic analysis techniques, many novel viruses have been discovered and isolated from arthropods over the recent past, but have not been registered in the Arbovirus Catalog^8,9^.

Section VIII-F of the Biosafety in Microbiological and Biomedical Laboratories (BMBL) 6th published by CDC in 2020 (https://www.cdc.gov/labs/BMBL.html), provides safety guidelines to those working with arbovirus, as well as ASV that are closely related to arboviral counterparts^10^. Table 3 and 4 in Section VIII-F of BMBL 6^th^ provides an alphabetical listing of the recognized arbovirus and ASV at the time of publication (by the year of 2019) and includes the common name, acronym, virus family or genus, Biosafety level (BSL) recommendation etc., separately.

International Committee on Taxonomy of Viruses (ICTV, https://ictv.global/) is aiming at categorizing viruses using a single classification method by their evolutionary relationships. The ICTV Virus Metadata Resource (VMR, VMR_18-191021_MSL36, https://ictv.global/vmr) provides virus meta information almost all classified viruses especially about the viruses classified after the year of 2019, but no specific information about if the virus belongs to arbovirus or ASV.

GenBank^11^ (https://www.ncbi.nlm.nih.gov/GenBank/) is a regularly updated public nucleotide sequence database, enlists many meta information of the virus as well as the nucleotide/amino acid sequences. However, the isolate, segment and host information of GenBank is complex and non-uniformly formatted due to the different standards adopted by the numerous submitters. In addition, it does not include categorical information on whether a virus is belonging to arbovirus or ASV.

Currently, there is no comprehensive dataset containing both arbovirus and ASV in a globally accessible scale. To address this issue, we collected, extracted, cleaned, and sorted information from the Arbovirus Catalog, Section VIII-F of the BMBL, ICTV and GenBank, that includes a complete information set on viral taxonomy, biological characteristics, vectors and vertebrate hosts, distribution, recommended biosafety levels, nucleotide/amino acid sequences, and genome segment. This dataset will be useful and beneficial to the larger community of scientists/researchers that study arbovirus and ASV, specifically in the fields of viral vector/host prediction through deep learning, disease outbreak risk warning, arbovirus/ASV interactions, phylogenetic and evolutionary relationships, as well as in biosafety risk assessment studies.

## Methods

### Date collection

In the first step, the virus names from Arbovirus Catalog/Table 3 and 4 of Section VIII-F of BMBL 6^th^ and Virus Metadata Resource_(18-191021_MSL36) from ICTV were extracted, then the records containing artificial (chimeric sequences, plasmid etc.) or short sequences (length <100 bp) were removed, to generate tempdata 1 and tempdata 2 separately. The corresponding submit/release date, taxonomy, isolate source were further extracted from the NCBI Taxonomy (https://ftp.ncbi.nih.gov/pub/taxonomy/) and NCBI Virus-Host (https://www.genome.jp/virushostdb/).

GenBank records belonging to the vertebrate virus (host source by vertebrate) in the tempdata 1 were excluded to generate tempdata 3.

To process the arbovirus and ASV which were not present in tempdata 3, we first extracted records with hosts derived from invertebrates only or from both invertebrates and vertebrates from tempdata 2 and removed records that were redundant from tempdata 3.

If the host source of them belonged to invertebrates or vertebrates, the virus associated with these records was selected and rechecked by relevant literature^2,12–25^ to ascertain if it was a true “arbovirus”. If the host source belonged to an invertebrate, a further check was done to see if the isolation source was hematophagous arthropods (keywords: ‘mosquito’, ‘aedes’, ‘culex’, ‘anopheles’, ‘ixodes’, ‘tick(s)’, ‘argas’, ‘midge’, ‘sandfly’), then identified as ‘ASV’. GenBank records identified as arbovirus and ASV in these steps were combined to generate tempdata 4.

Finally, tempdata 3 and 4 were combined to generate the viral meta information data of arbovirus and ASV with comprehensive information in Microsoft Excel 2019 spreadsheet (.xlsx), virus segment, biosafety recommendation, host, and other relevant information were subsequently added to the virus information file. Nucleotide and amino acid sequences were extracted from GenBank (downloaded on 28^th^ January 2023) according to viral meta information data by GenBank ID to generate the viral nucleotide sequences file (the complete viral genome or genomic fragments) in the fna (FASTA nucleic acid) format and amino acid sequences file in the faa (FASTA amino acid) format.

The final global dataset of viral sequence, diversity, distribution, and biosafety recommendation of arbovirus and ASV consist of one virus meta information file (.xlsx), one virus nucleotide sequence file (.fna), and one amino acid sequence file (.faa). The data screening and integration process was accomplished using software Pandas v2.0.0 (https://pandas.pydata.org/).

**Fig. 1:**
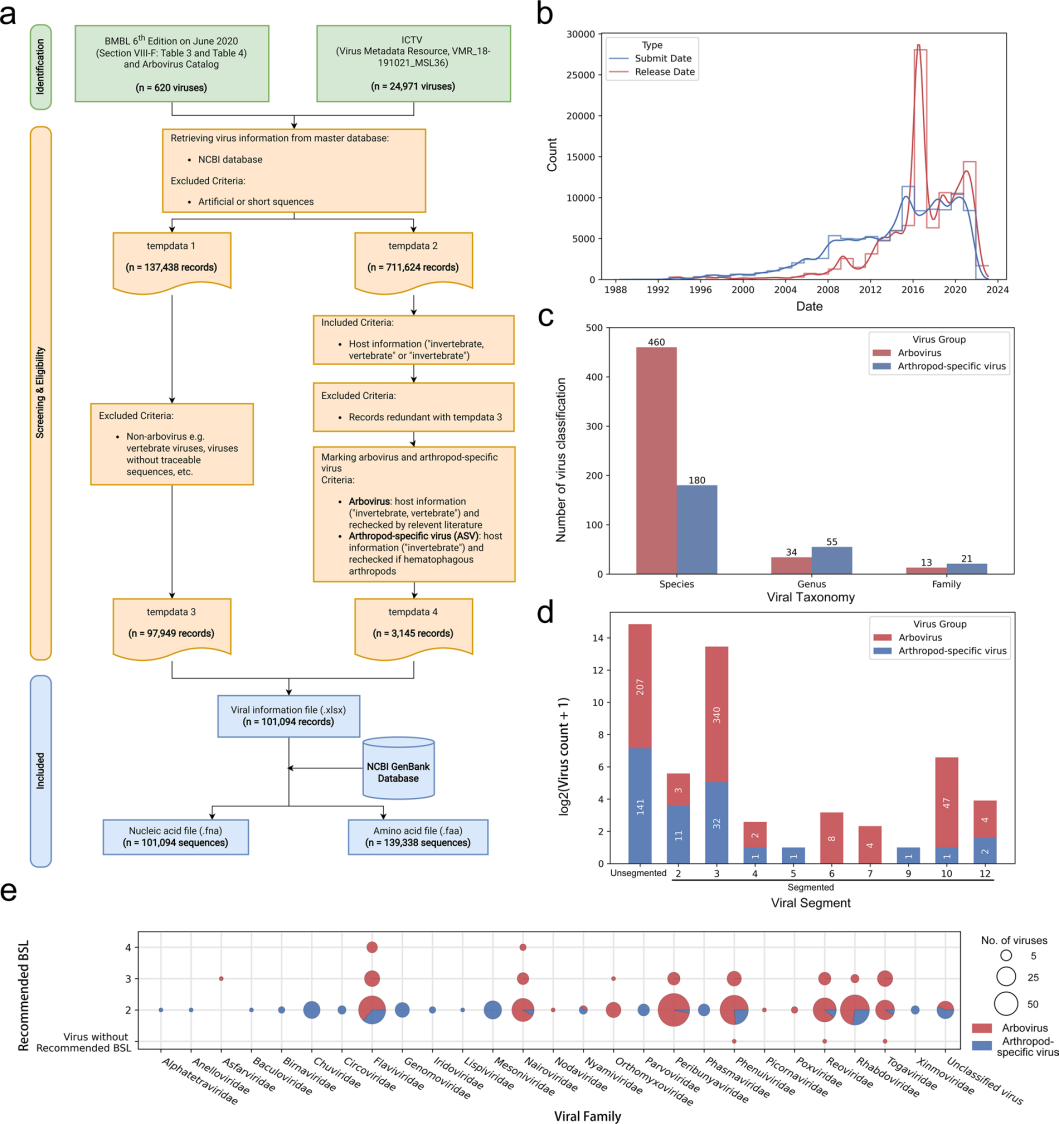
Schematic diagram of data process and statistics of the dataset. (**a**) Flow diagram on data collection, cleaning, and screening process. (**b**) Distribution of submission and release dates for the collected records retrieved from GenBank. (**c**) The number of families, genera and species of viruses in this dataset. (**d**) Distribution of arbovirus and arthropod-specific virus with different genomic segments in the dataset. (**e**) Distribution of recommended biosafety levels for arbovirus and arthropod-specific virus in different viral families. The red bar (or wedge) and blue bar (or wedge) represent arbovirus and arthropod-specific virus, respectively. *NAV, not available value.

A schematic view of the dataset construction is shown in (Fig. 1a).

**Fig. 2:**
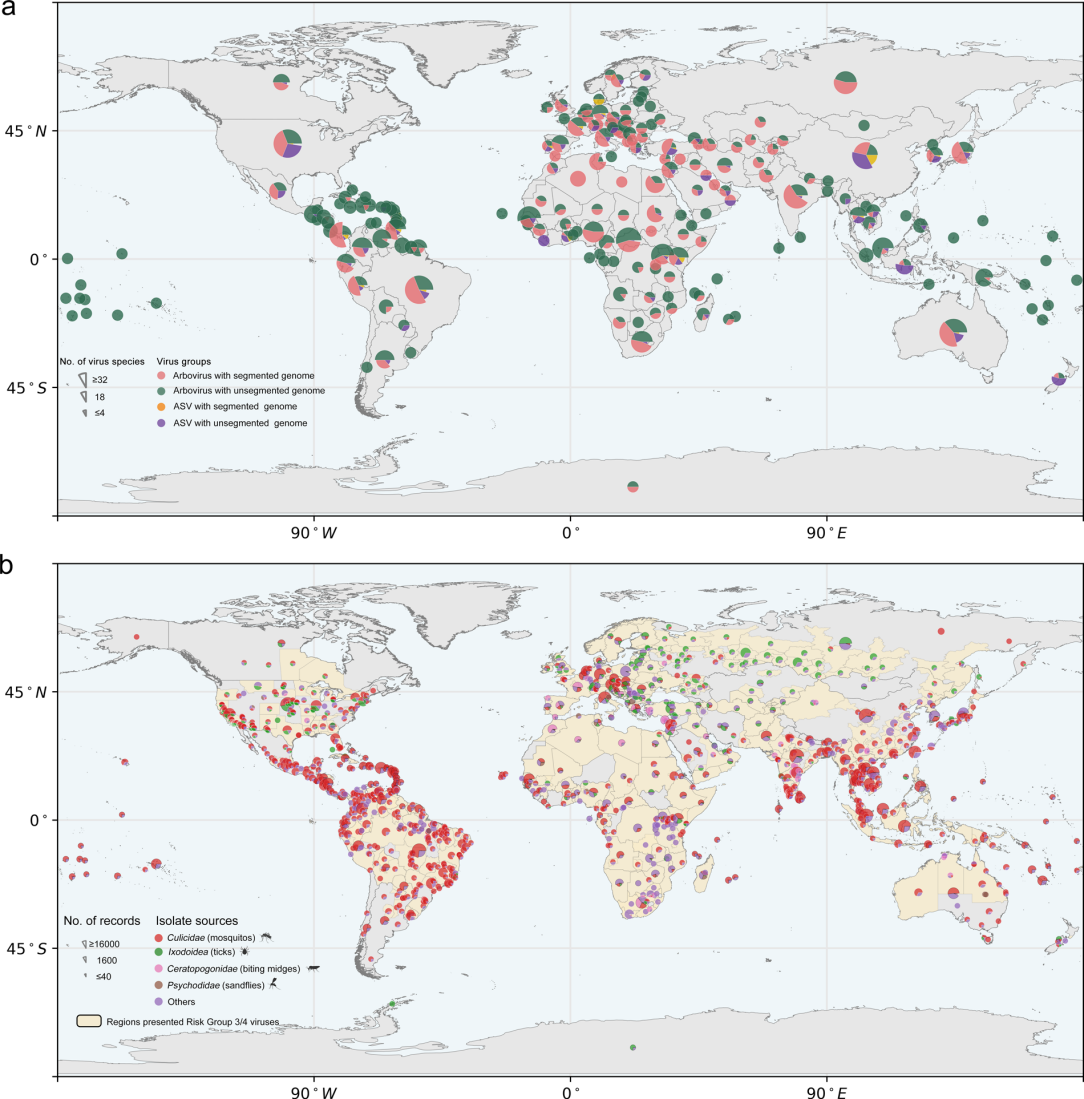
Geographical distribution of virus groups, vectors, genome segments, isolate sources and risk group in this dataset. (**a**) Distribution information of virus groups (arbovirus/arthropod-specific virus (ASV)) and genome segments (segmented/unsegmented) across the level of countries or regions. Each pie chart shows the percentages of arbovirus with segmented genome (pink), arbovirus with unsegmented genome (green), ASV with segmented genome (yellow), ASV with unsegmented genome (purple). The size of the pie chart represents the number of viruses species. (**b**) Distribution of isolate sources and risk group across the level of states/provinces (pie charts with black edges) and other more precise geographic locations. Each pie chart shows the percentages of *Culicidae* (red), *Ixodoidea* (green), *Ceratopogonidae* (pink), *Psychodidae* (brown) and other isolate sources (purple). The size of the pie chart represents the number of records. Areas previously reported presented the viruses belong to risk group 3 and 4 are highlighted with yellow.

A total of 620 virus names were derived from the Arbovirus Catalog/BMBL and 24,971 virus names were derived from the ICTV. Querying GenBank records based on these virus names and removing artificial sequences yielded 137,438 (tempdata 1) and 711,624 (tempdata 2) GenBank records, respectively. After removing records that were neither arbovirus nor ASV from tempdata1 and tempdata 2, 97,949 records (tempdata 3) and 3,145 (tempdata 4) records were used for dataset merging. Finally, a total of 101,094 eligible records (640 virus species/805 viruses) were generated, comprising 98,994 records (460 virus species/615 viruses) of arbovirus and 2,100 records (180 virus species/190 viruses) of ASV. The corresponding 101,094 viral nucleic acid sequences and 139,338 viral amino acid sequences were also extracted from the NCBI GenBank database (Fig. 1a). All records were submitted between the year 1988 to 2021 and released (or recently modified) from the year 1991 to 2022 (Fig. 1b). The viruses in this dataset belong to 34 families, 89 genera and 640 species (Fig. 1c). Information on virus segments and biosafety levels were also recorded (Fig. 1d,e). A profile of virus groups and genome segment numbers across countries or regions was displayed, as well as an overview of biosafety recommended levels and isolate sources distribution by geographic location (Fig. 2).

The most essential information extracted from the Arbovirus Catalog/BMBL, ICTV and NCBI database included (i) Locus ID and GenBank accession number, (ii) Taxonomy of viruses and their isolate sources, (iii) Segment number of the genome, (iv) Biosafety recommended level, and (v) Global geographical coordinates. All data was extracted and integrated into a final dataset including one excel sheet and two FASTA files, which were examined independently and thoroughly by a five-member team to avoid possible errors and redundancies.

### Geo-positioning

The geographical locations of all collected records were extracted from the NCBI GenBank database. This included countries or regions, states or provinces, latitude and longitude, and any other geographical information. All acquired geographical information was standardized to the countries or regions and state/province levels based on the digital map of the World Food and Agriculture Organization (FAO) (https://data.apps.fao.org/map/catalog/srv/eng/catalog.search#/metadata/9c35ba10-5649-41c8-bdfc-eb78e9e65654), with records without corresponding information populated with acronym ‘NAV’. The original latitude and longitude records and the centroids of countries/regions or states/provinces were used for the subsequent cartography. GeoPandas v0.12.2 (https://geopandas.org/en/stable/) and Matplotlib v3.6.0 (https://matplotlib.org/) were used for data processing and visualization of geographic information.

## Data Records

This global dataset of viral sequence, diversity, distribution, and biosafety recommendation for arbovirus and ASV contains a viral information file (.xlsx), a nucleic acid sequences file (.fna) and amino acid sequences file (.faa), as accessible from figshare^26^.

The column details of the viral meta information file (.xlsx) are as follows (The “NAV” in the field indicates not available value):

**Taxonomy Information**

1. ***Virus_Group*:** (customized field) viruses in the database are divided into two groups: arbovirus and ASV. The former has both vertebrate and arthropod hosts, the latter has only arthropod hosts.
2. ***Name*:** (source from GenBank) the virus name, each name represents a distinct virus.
3. ***Acronym*:** (source from BMBL) acronym of virus name.
4. ***NCBI_Taxonomy_ID*:** (source from GenBank) taxonomy identifier of virus from NCBI Taxonomy Database.
5. ***Isolate*:** (source from GenBank) Isolate of virus from NCBI GenBank.
6. ***Unified_Isolate_Number*:** (customized field) renumbering of the field *Isolate*. Each isolate of the same virus is numbered.
7. ***Species*:** (source from ICTV) species that the virus belongs to. Species of the viruses are normally different with their names.
8. ***Genus*:** (source from ICTV) genus that the virus belongs to.
9. ***Family*:** (source from ICTV) family that the virus belongs to.
**Genome Information**
10. ***Segmented*:** (customized field) whether the genome of the virus is unsegmented (recorded as “no”) or segmented virus (recorded as “yes”). Virus with an unknown number of segments (recorded as “NAV”).
11. ***Number_of_Segments*:** (source from GenBank) the theoretical number of segments of the virus.
12. ***Molecule_Type*:** (source from GenBank) molecule types of the virus genome which are divided into ssRNA(+), ssRNA(−), ssRNA(+/−), dsRNA, RNA, ssDNA(+/−), dsDNA and etc.
**Sequence Information**
13. ***Accession*:** (source from GenBank) NCBI GenBank Accession of the nucleotide sequence.
14. ***Locus*:** (source from GenBank) the locus name of the nucleotide sequence.
15. ***SRA_Accession*:** (source from GenBank) NCBI SRA Accession of the nucleotide sequence.
16. ***Submitters*:** (source from GenBank) submitters of the nucleotide sequence.
17. ***Sequence_Type*:** (source from GenBank) whether the nucleotide sequence is a reference sequence (recorded as “RefSeq”) or a non-reference sequence (recorded as “GenBank”).
18. ***BioSample*:** (source from GenBank) NCBI BioSample Accession of the nucleotide sequence.
19. ***GenBank_Title*:** (source from GenBank) the field “DEFINITION” of NCBI GenBank database of the sequence.
20. ***Genotype*:** (source from GenBank) genotype of the nucleotide sequence.
21. ***Segment*:** (source from GenBank) segment identifier of the nucleotide sequence.
22. ***Unified_Segment_Number*:** (customized field) renumbering of the field *Segment*. Each segment is assigned a new number from 1. Segment of the unsegmented virus is assigned as 1.
**Host Information**
23. ***Host_Species*:** (customized field) the species of the dead-end host of the virus.
24. ***Host_Genus*:** (customized field) the genus of the dead-end host of the virus.
25. ***Host_Family*:** (customized field) the family of the dead-end host of the virus.
26. ***Host*:** (source from GenBank) the field from the NCBI GenBank database that represents dead-end host or vectors.
**Biosafety Information**
27. ***Recommended_BSL*:** (customized field) recommended biosafety level of laboratory to research the virus (recorded as “2”, “3”, “4”, “NAV”).
28. ***BMBL_Recommended_BSL*:** (source from BMBL) BMBL recommended biosafety level of laboratory to research the virus (recorded as “2”, “2 with 3 practices”, “2b”, “3”, “3a”, “3b”, “4”, “NAV”).
29. ***Basis_of_Rating*:** (source from BMBL) risk assessment of the virus (recorded as “A1”, “A2”, “A3”, “A4”, “A7”, “IE”, “S”, “NAV”).
30. ***Antigenic_Group*:** (source from BMBL) the antigenic group of the virus.
31. ***Isolated*:** (customized field) whether the virus has been isolated (“Yes” or “No”).
**Source Information**
32. ***Latitude_and_Longitude*:** (source from GenBank) longitude and latitude of the virus isolation source.
33. ***State_or_Province*:** (customized field) state or provincial administrative unit of the virus source.
34. ***Geo_Location*:** (source from GenBank) geographical position of the virus source.
35. ***Country_or_Region*:** (customized field) the country or region of the virus source.
36. ***Isolation_Source*:** (source from GenBank) the organism which the virus was collected from.
37. ***Collection_Date*:** (source from GenBank) the date that the virus was collected.
38. ***Submit_Date*:** (source from GenBank) the date that the virus was submitted.
39. ***Release_Date*:** (source from GenBank) the date that the virus was released or last modified.
**References**
40. ***Publications*:** (customized field) the number of publications and literature covering the specific virus research.
41. ***Accession_URL*:** (customized field) the DOI leading directly to the GenBank source.

The nucleotide sequences file and amino acid sequences file are standard FASTA files. Each sequence information consists of two lines, header and content. The header contains two types of information, locus and accession, split by ‘|’. Content is a specific nucleic acid or amino acid sequence. The detailed definitions of the fields in the header are as follows:

1. ***Locus*:** (source from GenBank) NCBI GenBank LOCUS ID of the nucleotide sequence.
2. ***Accession*:** (source from GenBank) NCBI GenBank Accession of the nucleotide sequence.
3. ***Protein_ID*:** (source from GenBank) a protein sequence identification number (for amino acid sequences file).

## Technical Validation

This dataset collected 101,094 records (805 viruses) from 615 arboviruses and 190 ASVs derived from 849,062 GenBank records (25,591 viruses), that were submitted and released from 1988 to 2022. All records were collected and processed by two members of the team with the other three members tasked with cross-checking and confirming, and all uncertain or discrepant records were discussed separately.

**Fig. 3:**
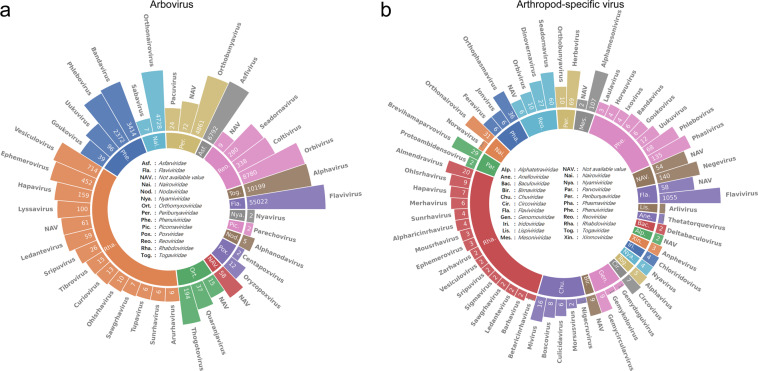
Viral taxonomic composition of arbovirus and arthropod-specific virus in this dataset. The nested pie charts show the percentage of (**a**) arbovirus and (**b**) arthropod-specific virus at the family (inner circle) and genus (outer circle) levels. The length of the bars in the outer circle indicates the corresponding number of records, where detailed entries are marked. *NAV, not available value.

**Fig. 4:**
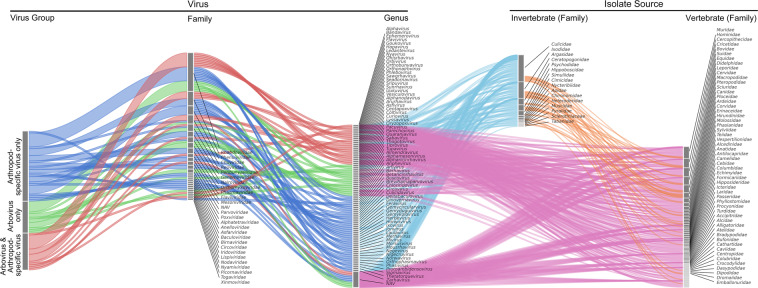
The correspondence between taxonomy and isolate source of viruses in this dataset at the genus level. All viral genera are divided into three groups according to the virus groups, including both arbovirus and arthropod-specific virus (red), arbovirus only (blue), and arthropod-specific virus only (green). The isolate sources of all viruses are divided into both vertebrate and invertebrate (orange), vertebrate only (pink), and invertebrate only (blue). *NAV, not available value.

The accuracy of virus group classification (arbovirus/ASV) is critical, therefore the virus groups of records in this database were first defined by referring to Tables 3 and 4 in Section VIII-F of the BMBL 6^th^ and Arbovirus Catalog, a reliable source for record arbovirus and ASV records. Other records that were not queried in the Arbovirus Catalog/BMBL 6^th^ were manually inspected against relevant literature and isolate sources. To ensure the accuracy and reliability of manual inspection, the following strategies were adopted: (i) If the isolate source contained both vertebrates and invertebrates, the specific host information reported in the relevant literature for the record was used to determine the virus type (arbovirus, ASV or others), (ii) If the isolate source contains invertebrates only and belongs to hematophagous arthropods (*Culicidae, Ixodoidea, Ceratopogonidae, Phlebotominae bailinyake, Tabanidae, Hippoboscidae*), the record was labelled as ASV. The taxonomy at the family and genus level of all included arbovirus and ASV, as well as the correspondence between virus taxonomy and isolate sources, were shown in Figs. 3, 4. Due to the diversity of geographic names in GenBank records, the geographic names in countries or regions and states or provinces in the FAO digital maps were manually re-searched in GenBank records to retrieve as many matching geographic names as possible.

## Usage Notes

Emerging and re-emerging viral diseases transmitted by arthropods remain one of the most serious threats to humans and animals, with potentially devastating social and economic consequences. Knowledge of the classification, molecular characteristics of their genomes, abundance and diversity, worldwide distribution, vector and vertebrate host, and biosafety risk of arbovirus and ASV is crucial in supporting the development of policies and directing the necessary actions for preventing and managing relevant diseases, and developing novel biological control strategies. This dataset serves as the foremost comprehensive compilation of both arbovirus and ASV on a global scale, which can be used in biosafety risk assessment, to infer arbovirus and ASV evolutionary relationships, model arboviral diseases’ ecological risks, and infer vectors and hosts of the newly discovered arbovirus.

## Data Availability

No custom code was made for the compilation and validation procedures in this dataset.
